# Relationship between fetal middle cerebral artery pulsatility index and cerebroplacental ratio with adverse neonatal outcomes in low-risk pregnancy candidates for elective cesarean section: A cross-sectional study

**DOI:** 10.18502/ijrm.v20i8.11755

**Published:** 2022-09-06

**Authors:** Fatemeh Golshahi, Behrokh Sahebdel, Mahboobeh Shirazi, Fatemeh Rahimi Sharbaf, Hossein Rezaei Aliabadi, Mona Taghavipour, Seyede Houra Mousavi Vahed, Tayebeh Sedighi Darijani, Maria Nezamnia

**Affiliations:** ^1^Maternal, Fetal, and Neonatal Research Center, Yas Complex Hospital, Tehran University of Medical Sciences, Tehran, Iran.; ^2^School of Medicine, Bam University of Medical Sciences, Bam, Iran.; ^3^Department of Obstetrics and Gynecology, Mashhad University of Medical Sciences, Mashhad, Iran.; ^4^School of Medicine, Ayatollah Kashani Hospital, Jiroft University of Medical Sciences, Jiroft, Iran.

**Keywords:** Umbilical cord blood, Color Doppler ultrasonography, Gestational age.

## Abstract

**Background:**

The cerebroplacental ratio (CPR) is an important factor for predicting adverse neonatal outcomes in appropriate-for-gestational-age fetuses.

**Objective:**

To evaluate whether there is an association between the CPR level and adverse neonatal outcomes in appropriate-for-gestational-age fetuses.

**Materials and Methods:**

This cross-sectional study included 150 low-risk pregnant women candidates for elective cesarean sections at the gestational age of 39 wk. CPR and middle cerebral artery pulsatility index (MCA PI) were calculated in participants just before cesarian section. Postnatal complications were defined as an adverse neonatal outcome such as an Apgar score of the neonate ≤ 7 at 5 min, neonatal intensive care unit (NICU) admission, cord arterial pH ≤ 7/14, and meconium stained liquor.

**Results:**

The mean age of participants was 31.53 ± 4.91 yr old. The mean CPR was reported as 1.83 ± 0.64. The Chi-square test analysis revealed that a low MCA PI and a low CPR were significantly associated with decreased cord arterial pH, decreased Apgar score at 5 min, and NICU admission (p < 0.001). There was no significant association between umbilical artery PI with arterial cord pH, Apgar score at 5 min, NICU admission, or meconium stained liquor. The Mann-Whitney test showed that a lower fetal weight appropriate for the women's gestational age was significantly associated with a decreased CPR and MCA PI (p < 0.005). There was no significant association between amniotic fluid index and CPR, umbilical artery PI, or MCA PI.

**Conclusion:**

The CPR is a significant factor in predicting adverse neonatal outcomes and ultimately neonatal mortality and morbidity of low risk, appropriate-for-gestational-age fetuses.

## 1. Introduction

Adverse neonatal outcomes are among the greatest challenges for obstetrics (1). Fetal hypoxia occurs for a variety of reasons and might lead to neonatal mortality and morbidity. Numerous conditions such as maternal infections, medical disorders, preterm delivery, maternal bleeding, and fetal growth restriction can cause hypoxia and fetal distress (2-5). Decreased fetal cerebral blood flow leads to activation of compensatory mechanisms by reducing the resistance of cerebral arteries to supply cerebral oxygenation (5, 6). Fetal hypoxia is assessed using different tests such as the non-stress test, amniotic fluid index (AFI), biophysical profile, and fetal vascular Doppler ultrasound (2, 7, 8). The middle cerebral artery pulsatility index (MCA PI) and umbilical artery pulsatility index (UA PI) are used to evaluate fetal hypoxia with Doppler ultrasound. The cerebroplacental ratio (CPR) is also a significant predictor in the evaluation of hypoxia and fetal distress (8).

Due to uterine contractions during normal vaginal delivery (NVD), the blood supply to the fetal arteries might be affected, which causes the fetus to be exposed to varying degrees of hypoxia and perinatal complication (9). A study showed that a low CPR reflects fetal hypoxemia (e.g. brain sparing) in fetal growth restriction (FGR) and helps to identify adverse neonatal outcomes (10). In 2018 a study showed that a lower CPR is associated with a higher risk of intrapartum fetal distress and composite adverse perinatal outcomes (11). Studies have been performed in recent years to investigate the relationship between fetal vascular Doppler indices and the increased risk of adverse neonatal outcomes in NVD candidates as well as in FGR cases and showed that a low MCA PI was associated with an increased risk of adverse neonatal outcomes (12-14). In the present study, the confounding factors of NVD and FGR were eliminated as only candidates for elective cesarean section (C-section) with fetal weight appropriate for gestational age (AGA) were included.

This study aimed to explore the relationship between fetal MCA PI and CPR with adverse neonatal outcomes in low-risk pregnancies, as well as the accuracy of CPR in predicting mortality and morbidity in infants.

## 2. Materials and Methods

This cross-sectional study was performed from November 2020 to November 2021 with 150 pregnant women referred to Yas University hospital (Tehran University of Medical Sciences, Tehran, Iran).

The inclusion criteria were: singleton pregnant women with a gestational age of 39 wk, fetal weight in AGA, and admitted for elective C-section due to previous C-section or breech presentation. The exclusion criteria were: history of maternal underlying disease or condition (such as diabetes mellitus, hypertension, vascular disease, thyroid disease), FGR, fetal abnormalities, rupture of membranes, maximum vertical pocket < 2 cm, and multiple gestations.

A questionnaire was used to collect data on each hospitalized pregnant woman which included demographic, para-clinical, and clinical information of candidates. Pregnant hospitalized women who did not meet the exclusion criteria were defined as low-risk pregnancies and were evaluated. All routine physical examinations, lab tests, and ultrasounds were performed, and the gestational age was calculated based on the Nuchal translucency ultrasonography. Half an hr before C-section, women underwent an ultrasound to assess fetal weight, AFI, and fetal vascular Doppler. All ultrasounds were performed by a perinatologist by using an ultrasound device (Philips, Affinity 50, USA), with a 6 MHz probe. For each of the pregnant women, the AFI was measured by ultrasound and the cutoff level for AFI was determined as 5 cm. Women with a maximum vertical pocket < 2 cm were excluded from the study. An AFI level ≤ 5 cm was classified as oligohydramnios and 5-24 cm was considered normal (15). Fetal weight determination by ultrasound was measured according to the Hadlock table with 4 parameters: biparietal diameter, head circumference, femoral length, and abdomen circumference.

Moreover, if the fetal weight and abdomen circumference were < 10 ${}^{\text{th}}$ percentile, the pregnant mother was excluded from the study with an FGR diagnosis. Full-term infants with a weight between the 10 ${}^{\text{th}}$ -90 ${}^{\text{th}}$ percentile were considered AGA (14). Vascular Doppler parameters including MCA PI, UA PI, and CPR were calculated and recorded in each data sheet. In fetal vascular examination, pulsed Doppler parameters were automatically calculated from 3 or more consecutive waveforms using a convex probe, with an irradiation angle of approximately 0 , during fetal quiescence and during the absence of fetal tachycardia. Doppler recording of the fetal middle cerebral artery (near the Willis ring and at the point where the sphenoid wing intersects with the artery) was examined.

Additionally, Doppler data of the umbilical artery was also recorded in a free umbilical cord ring. CPR was calculated according to the formula of MCA to UA PI ratio. According to the International Society of Ultrasound in Obstetrics and Gynecology 2005 guideline, for accurate measurement in vascular Doppler, the cutoff level was defined at 39 wk of gestation. According to the International Society of Ultrasound in Obstetrics and Gynecology 2005 guideline, the cutoff level defined in CPR was ≤ 1.29 ( < 5 ${}^{\text{th}}$ percentile) and > 1.29 ( > 5 ${}^{\text{th}}$ percentile); in MCA PI ≤ 1.10 ( < 5 ${}^{\text{th}}$ percentile) and > 1.10 ( > 5 ${}^{\text{th}}$ percentile); and in UA PI ≤ 1.10 ( < 95 ${}^{\text{th}}$ percentile) and > 1.10 ( > 95 ${}^{\text{th}}$ percentile) (16).

If the numbers obtained for CPR and MCA PI were less than or equal to the cutoff level, the women were put in the abnormal group. Women were also allocated to the abnormal group if their UA PI numbers were greater than the defined cutoff level. After performing vascular Doppler ultrasound, the pregnant women underwent C-sections and the gynecologists who performed the C-sections were blinded to the findings of the vascular Doppler.

Finally, postnatal complications were defined as an adverse neonatal outcome and included:

• Apgar score of the neonate ≤ 7 at 5 min

• The neonates' arterial blood pH ≤ 7/14

• Neonatal intensive care unit (NICU) admission

• Meconium stained liquor

Infant weight was measured postnatally to eliminate the possibility of sonographic error and was recorded in a checklist for each pregnant mother.

### Ethical considerations

This study was approved by the Ethics Committee of Tehran University of Medical Sciences, Tehran, Iran (Code: IR.TUMS.MEDICINE.REC.1400.1099). All women provided informed consent prior to the study.

### Statistical analysis

For this study, a sample size of 150 participants with low risk pregnancies who were candidates for elective C-section was chosen. After reviewing the normality test, we used non-parametric methods to investigate the relationship between the variables. The Mann-Whitney test was used to examine the relationship between variables for which the normality test was not established. In addition, we used the Chi-square test to analyze the association among variables. The significance level was determined as 0.01, and all the above-mentioned analyses were performed using Statistical Package for Social Sciences v. 19 (SPSS, Inc, Chicago, IL).

## 3. Results 

The demographic details of the participants are summarized in table I. Out of the total 150 participants, the mean age of the participants was 31.53 ± 4.91 yr old. The minimum age was 21 yr old and the maximum age was 42 yr old. The mean CPR was 1.83 ± 0.64, the minimum CPR was 0.83, and the maximum CPR was 3.82. It was shown that 32 (21.3%) of the participants had a CPR ≤ 1.29 ( < 5 ${}^{\text{th}}$ percentile), and 118 (78.7%) had a CPR > 1.29 ( > 5 ${}^{\text{th}}$ percentile). Furthermore, 23 (15.3%) of the participants had an MCA PI ≤ 1.10 ( < 5 ${}^{\text{th}}$ percentile) and 127 (84.7%) had an MCA PI > 1.10 ( > 5 ${}^{\text{th}}$ percentile).

In the UA PI analysis, it was found that 119 (79.3%) of the participants had a UA PI ≤ 1.10 ( < 95 ${}^{\text{th}}$ percentile), and 31 (20.7%) had a UA PI > 1.10 ( > 95 ${}^{\text{th}}$ percentile). In this study, 17 (11.3%) participants had a cord arterial pH ≤ 7.14, and 133 (88.7%) had a cord arterial PH > 7.14. Also, 14 (9.3%) of the newborns had an Apgar score of ≤ 7 at 5 min and 13 (8.7%) of the newborns were admitted to the NICU.

The Chi-square analysis revealed that a low CPR was significantly associated with decreased cord arterial pH, decreased Apgar score at 5 min, and NICU admission (p < 0.001) (Table II). As shown in table II, there was no significant association between UA PI and arterial cord pH, Apgar score at 5 min, NICU admission, or meconium stained liquor. Also, table II shows that decreased cord arterial pH, decreased Apgar score at 5 min, and NICU admission were significantly associated with a low MCA PI (p < 0.001). The Mann-Whitney test results shown in table III revealed that fetal weight in AGA was significantly associated with a low CPR and MCA PI (p < 0.005). Table IV shows that there was no significant association between AFI and CPR, UA PI, or MCA PI.

**Table 1:** Demographic characteristics of the participants


**Variables**	**Mean ± SD**	**Min**	**Max**
**Age (yr)**	31.53 ± 4.91	21	42
**Amniotic fluid index **	13.06 ± 4.38	4	30
**Umbilical artery pulsatility index**	0.91 ± 0.19	0.45	1.25
**Middle cerebral artery pulsatility index**	1.59 ± 0.44	0.85	2.82
**Cerebroplacental ratio**	1.83 ± 0.64	0.83	3.82
SD: Standard deviation, Min: Minimum, Max: Maximum			

**Table 2:** Association between cerebroplacental ratio, UA PI, and MCA PI with cord pH, Apgar score at 5 min, meconium stained liquor, and neonatal intensive care unit admission


**Variables**		**Cord pH**		**Apgar score at 5 min**		**Meconium stained liquor**		**NICU admission**	
		**≤ 7.14**	**> 7.14**	**≤ 7**	**> 7**	**Yes**	**No**	**Yes**	**No**
**Cerebroplacental ratio**									
	**≤ 1.29**	13 (76.5)	19 (14.3)	12 (85.7)	20 (14.7)	1 (16.7)	31 (21.5)	10 (76.9)	22 (16.1)
	**> 1.29**	4 (23.5)	114 (85.7)	2 (14.3)	116 (85.3)	5 (83.3)	113 (78.5)	3 (23.1)	115 (83.9)
P-value*		< 0.001 ${}^{\#}$		< 0.001 ${}^{\#}$		0.77		< 0.001 ${}^{\#}$	
**Umbilical artery pulsatility index**									
	** ≤ 1.10**	11 (64.7)	108 (81.2)	8 (57.1)	111 (81.6)	5 (83.3)	114 (79.2)	10 (76.9)	109 (79.6)
	** > 1.10**	6 (35.3)	25 (18.8)	6 (42.9)	25 (18.4)	1 (16.7)	30 (20.8)	3 (23.1)	28 (20.4)
P-value*		0.11		0.03		0.98		0.82	
**Middle cerebral artery pulsatility index**									
	**≤ 1.10**	8 (47.1)	15 (11.3)	7 (50.0)	16 (11.8)	1 (16.7)	22 (15.3)	8 (61.5)	15 (10.9)
	**> 1.10**	9 (52.9)	118 (88.7)	7 (50.0)	120 (88.2)	5 (83.3)	122 (84.7)	5 (38.5)	122 (89.1)
P-value*		< 0.001 ${}^{\#}$		< 0.001 ${}^{\#}$		0.52		< 0.001 ${}^{\#}$	
Data presented as n (%). ${}^{*}$ Chi-square test, ${}^{\#}$ P < 0.01. NICU: Neonatal intensive care unit									

**Table 3:** Association between fetal weight in appropriate for gestational age with cerebroplacental ratio, umbilical artery pulsatility index and middle cerebral artery pulsatility index


**Variables**		**Median (IQR)**	P-value ${}^{*}$
**Cerebroplacental ratio**			
	** ≤ 1.29**	2800.0 (231.2)	
	** > 1.29**	3000.0 (407.5)	< 0.001 ${}^{\#}$
**Umbilical artery pulsatility index**			
	**≤ 1.10**	2950.0 (350.0)	
	**> 1.10**	2960.0 (350.0)	0.71
**Middle cerebral artery pulsatility index**			
	**≤ 1.10**	2800.0 (300.0)	
	**> 1.10**	3000.0 (350.0)	< 0.001 ** ${}^{\#}$ **
${}^{*}$ Mann-Whitney test, ${}^{\#}$ P < 0.01, IQR: Inter quartile range			

**Table 4:** Association between amniotic fluid index with cerebroplacental ratio, umbilical artery pulsatility index, and middle cerebral artery pulsatility index


**Variables**		**Amniotic fluid index**		P-value ${}^{*}$
		**> 5**	**≤ 5**	
**Cerebroplacental ratio**				
	**≤ 1.29**	32 (100.0)	0 (0)	
	**> 1.29**	113 (95.8)	5 (4.2)	0.58
**Umbilical artery pulsatility index**				
	**≤ 1.10**	115 (96.6)	4 (3.4)	
	**> 1.10**	30 (96.8)	1 (3.2)	0.97
**Middle cerebral artery pulsatility index**				
	**≤ 1.10**	21 (91.3)	2 (8.7)	
	**> 1.10**	124 (97.6)	3 (2.4)	0.12
Data presented as n (%). ${}^{*}$ Chi-square test				

## 4. Discussion

CPR is an important factor in predicting adverse neonatal outcomes and ultimately neonatal morbidity and mortality. In this study, we observed the association between CPR and adverse neonatal outcomes in AGA fetuses. The results of this study demonstrated that lower CPR and MCA PI levels are associated with increased adverse neonatal outcomes which lead to an increase in NICU admission, neonatal acidosis, and decreased Apgar score at 5 min. According to these findings, a lower CPR can be a predictor for adverse neonatal outcomes even in AGA fetuses.

In terms of meconium stained liquor, which was one of the adverse neonatal outcomes, decreased CPR and MCA PI levels were unrelated. This could be explained by the small number of meconium stained liquor patients due to regular monitoring in the third trimester of pregnancy.

Also, a higher fetal UA PI was not associated with adverse neonatal outcomes. This could be explained by an associated increase in cerebrovascular compensatory flow occurring by decreasing the fetal cerebral vascular resistance. Fetal hypoxia and neonatal adverse outcomes usually occur in the third trimester of pregnancy following a decrease in resistance to MCA PI that leads to a decrease in CPR. Such a decrease in CPR may be independent of changes in UA PI.

In this study, the relationship between CPR and MCA PI with median weight in the AGA fetuses was investigated, showing that a lower fetal weight in the AGA fetuses was associated with a lower CPR and MCA PI, suggesting that maybe these fetuses, although not FGR, had not reached their growth potential. Fetuses with a weight between the 10 ${}^{\text{th}}$ and 90 ${}^{\text{th}}$ percentile are considered AGA. Despite being healthy, some AGA fetuses may have placental insufficiency (probably those who have not reached their growth potential) which may lead to adverse neonatal outcomes. Based on these findings, it can be inferred that lower fetal weight, even within the AGA range, is associated with adverse neonatal outcomes.

Moreover, no significant relationship was found between oligohydramnios and CPR or MCA PI in this study. This can be explained by the careful monitoring that occurred in the third trimester of pregnancy.

Numerous studies have shown that a low CPR indicates redistribution of fetal blood flow according to the brain-sparing hypothesis and predicts adverse neonatal outcomes (17-19). Some studies have shown that CPR is a major independent predictor of stillbirth and perinatal morbidity. Even in low-risk pregnancies candidates for NVD, a low CPR increases the risk of C-section (20-22).

In 2015, a study showed that CPR had a high sensitivity in the prediction of fetal heart rate abnormalities and adverse neonatal outcomes in low-risk pregnancies at 40 wk and beyond (23). Also, in 2020 a study showed that a lower CPR in AGA fetuses was associated with a higher risk of C-section and adverse neonatal outcomes which is similar to the results of our study. Our study differed in that we had a larger sample size and eliminated the NVD confounding variable (24). Another study conducted in 2021 found that a lower CPR in AGA fetuses at 37-40 wk of gestation was associated with a higher risk of C-section and adverse neonatal outcomes (25). Therefore, a low CPR in AGA fetuses can be a sign of hypoxia and adverse neonatal outcomes, although further studies are needed to confirm this (26).

The strength of this prospective study lies in its design which minimized the impact of any confounding factors by only including low-risk individuals. Moreover, only women undertaking an elective C-section were included to remove the confounding effect of vascular Doppler changes during NVD. Additionally, Color Doppler was performed by an experienced perinatologist. The cut-off level was defined as the lowest possible level for the sensitivity of vascular Doppler findings, and the gynecologists who performed the C-section were blinded to the vascular Doppler findings.

A low CPR in AGA fetuses was associated with an increased risk of adverse neonatal outcomes, a lower neonatal cord arterial pH and Apgar score, and a higher risk of NICU admission. Therefore, based on these findings, it is recommended that CPR is checked in women candidates for elective C-section at 38 wk of gestation. If the CPR is < 5%, a C-section at 38 wk of gestation may be helpful in the presence of a neonatologist to prevent adverse neonatal outcomes. However, more research is needed with a larger sample size to conclude whether earlier termination of pregnancy would be cost-effective and safe in these cases.

## 5. Conclusion

CPR is a non-invasive important factor in predicting adverse neonatal outcomes and ultimately neonatal mortality and morbidity. Even in AGA fetuses, a low CPR can predict adverse neonatal outcomes.

##  Conflict of Interest

The authors declare that there is no conflict of interest.
